# Recommendations for neonatologists and pediatricians working in first level birthing centers on the first communication of genetic disease and malformation syndrome diagnosis: consensus issued by 6 Italian scientific societies and 4 parents’ associations

**DOI:** 10.1186/s13052-021-01044-1

**Published:** 2021-04-19

**Authors:** Gregorio Serra, Luigi Memo, Alessandra Coscia, Mario Giuffré, Ambra Iuculano, Mariano Lanna, Diletta Valentini, Anna Contardi, Sauro Filippeschi, Tiziana Frusca, Fabio Mosca, Luca A. Ramenghi, Corrado Romano, Annalisa Scopinaro, Alberto Villani, Giuseppe Zampino, Giovanni Corsello, on behalf of their respective Scientific Societies and Parents’ Associations

**Affiliations:** 1 Department of Health Promotion, Mother and Child Care, Internal Medicine and Medical Specialties “G. D’Alessandro”, University of Palermo, Palermo, Italy; 2 Clinical Genetics Outpatient Service, Neonatology and Neonatal Intensive Care Unit, San Bortolo Hospital, Vicenza, Italy; 3 University Neonatology Unit, AOU Città della Salute e della Scienza, Turin, Italy; 4 Unit of Prenatal and Preimplantation Diagnosis, Thalassaemic Hospital, AO Brotzu, Cagliari, Italy; 5 Unit of Obstetrics and Gynecology, Prenatal Diagnosis and Fetal Therapy “U. Nicolini”, Buzzi Hospital, ASST FBF Sacco, Milan, Italy; 6 Unit of General Pediatrics, Emergency and Acceptance Department, Bambino Gesù Pediatric Hospital, Rome, Italy; 7 Coordinator of the Italian Association of Down People, Rome, Italy; 8 President of the Italian National Association of Volunteers Cornelia de Lange, Pesaro, Italy; 9 President of the Italian Society of Obstetric and Gynecological Ultrasound and Biophysical Methodologies, Parma, Italy; 10 President of the Italian Society of Neonatology, Milan, Italy; 11 President of the Italian Society of Perinatal Medicine, Genoa, Italy; 12 Coordinator of the Clinical Genetics Study Group of the Italian Society of Human Genetics, Troina, EN Italy; 13 President of Italian Federation of Rare Diseases and of Williams Syndrome People Association, Rome, Italy; 14 President of the Italian Society of Pediatrics, Rome, Italy; 15 President of the Italian Society of Pediatric Genetic Diseases and Congenital Disabilities, Rome, Italy

**Keywords:** Genetic disease, Malformation syndrome, Communication of diagnosis, Consensus, Recommendations

## Abstract

**Background:**

Genetic diseases are chronic conditions with relevant impact on the lives of patients and their families. In USA and Europe it is estimated a prevalence of 60 million affected subjects, 75% of whom are in developmental age. A significant number of newborns are admitted in the Neonatal Intensive Care Units (NICU) for reasons different from prematurity, although the prevalence of those with genetic diseases is unknown. It is, then, common for the neonatologist to start a diagnostic process on suspicion of a genetic disease or malformation syndrome, or to make and communicate these diagnoses. Many surveys showed that the degree of parental satisfaction with the methods of communication of diagnosis is low. Poor communication may have short and long-term negative effects on health and psychological and social development of the child and his family. We draw up recommendations on this issue, shared by 6 Italian Scientific Societies and 4 Parents’ Associations, aimed at making the neonatologist’s task easier at the difficult time of communication to parents of a genetic disease/malformation syndrome diagnosis for their child.

**Methods:**

We used the method of the consensus paper. A multidisciplinary panel of experts was first established, based on the clinical and scientific sharing of the thematic area of present recommendations. They were suggested by the Boards of the six Scientific Societies that joined the initiative: Italian Societies of Pediatrics, Neonatology, Human Genetics, Perinatal Medicine, Obstetric and Gynecological Ultrasound and Biophysical Methodologies, and Pediatric Genetic Diseases and Congenital Disabilities. To obtain a deeper and global vision of the communication process, and to reach a better clinical management of patients and their families, representatives of four Parents’ Associations were also recruited: Italian Association of Down People, Cornelia de Lange National Volunteer Association, Italian Federation of Rare Diseases, and Williams Syndrome People Association. They worked from September 2019 to November 2020 to achieve a consensus on the recommendations for the communication of a new diagnosis of genetic disease.

**Results:**

The consensus of experts drafted a final document defining the recommendations, for the neonatologist and/or the pediatrician working in a fist level birthing center, on the first communication of genetic disease or malformation syndrome diagnosis. Although there is no universal communication technique to make the informative process effective, we tried to identify a few relevant strategic principles that the neonatologist/pediatrician may use in the relationship with the family. We also summarized basic principles and significant aspects relating to the modalities of interaction with families in a table, in order to create an easy tool for the neonatologist to be applied in the daily care practice. We finally obtained an intersociety document, now published on the websites of the Scientific Societies involved.

**Conclusions:**

The neonatologist/pediatrician is often the first to observe complex syndromic pictures, not always identified before birth, although today more frequently prenatally diagnosed. It is necessary for him to know the aspects of genetic diseases related to communication and bioethics, as well as the biological and clinical ones, which together outline the cornerstones of the multidisciplinary care of these patients. This consensus provide practical recommendations on how to make the first communication of a genetic disease /malformation syndrome diagnosis. The proposed goal is to make easier the informative process, and to implement the best practices in the relationship with the family. A better doctor-patient/family interaction may improve health outcomes of the child and his family, as well as reduce legal disputes with parents and the phenomenon of defensive medicine.

## Background

Genetic diseases are chronic conditions with relevant impact on the lives of patients and their families. Their clinical presentation is heterogeneous, and include: malformation syndromes which may be easily recognized (i.e. Down syndrome); malformation syndromes with evident phenotypes, but uncertain diagnosis only on clinical ground; suspected genetic disorders without evident malformations, which however involve organs and/or systems. In USA and Europe, it is estimated a prevalence of 60 million affected subjects (with an incidence of major malformations of 3% on the total of newborns), 75% of whom are in developmental age (in about a quarter of cases the diagnosis of such pathologies is made and communicated after the pediatric age) [1, 2]. A significant number of newborns are admitted in the Neonatal Intensive Care Units (NICU) for reasons different than prematurity, although the prevalence of those with genetic diseases is unknown [1]. It is common, therefore, for the neonatologist to start a diagnostic process on the suspicion of a genetic disease or malformation syndrome, or to make and communicate these diagnoses. He is often the first to inform parents, and communication is even more difficult when such news is unexpected. The communication of diagnosis is part of the daily work of the neonatologist and pediatrician and must be considered a medical act in all respects, like any other diagnostic or therapeutic procedure. For the neonatologist and the pediatrician of a birthing center, informing parents that their baby is affected by a malformation syndrome is one of the most delicate and difficult experiences. The methods of communication of diagnosis and the content of the information provided are decisive for parents to control their emotions, ease the painful effect of the news and find resources to face the future. Many surveys assessed the degree of parental satisfaction with the methods of communication of diagnosis [3, 4]. They showed that dissatisfaction is avoidable, and that it is not linked to the condition object of the communication, but to the modalities in which it is demonstrated and explained [3]. Moreover, poor communication may cause distrust and aversion towards doctors, and prevent good cooperation between family and health care professionals. It has a long-term negative effect both on the ability to accept the diagnosis and to adapt to the new situation, and on the developing relationships between parents and children. Health and psychological and social development of the child may be, then, adversely affected for a long time. In the international literature specific guidelines on this topic are few, and most are not recent [5–9]. We draw up recommendations on this issue, shared by 6 Italian Scientific Societies and 4 Parents’ Associations, aimed at making the neonatologist’s task easier, at the time of communication to parents of a genetic disease/malformation syndrome diagnosis for their child. The goal is also to reassure families on the most suitable care pathway, avoid raising inappropriate expectations and, at the same time, optimize their resources (psychological, cultural, socioeconomic) and implement skills and competences of their child.

## Methods

We used the method of the consensus paper [10, 11]. A multidisciplinary panel of experts was first established, based on the clinical and scientific sharing of the thematic area which is object of the recommendations.

Doctors with specific expertise and experience, acquired in communicating a genetic disease diagnosis, were identified. They were suggested by the Boards of the six Scientific Societies which joined the initiative: Italian Societies of Pediatrics, Neonatology, Human Genetics, Perinatal Medicine, Obstetric and Gynecological Ultrasound and Biophysical Methodologies, and Pediatric Genetic Diseases and Congenital Disabilities. To obtain a deeper and global vision of the communication process, and to reach a better clinical management of patients and their families, representatives of four Parents’ Associations were also recruited: Italian Association of Down People, Cornelia de Lange National Volunteer Association, Italian Federation of Rare Diseases, and Williams Syndrome People Association. They worked from September 2019 to November 2020 to achieve a consensus on the recommendations for the communication of a new diagnosis of genetic disease.

Initially, a systematically revision of the literature was made, inserting the terms “communication”, “diagnosis”, “genetic/genomic disease”, “congenital malformation”, “newborn” in the PubMed search engine. Papers published in the last 30 years (from 1991 to date) were analyzed, also relating to thematic areas different from clinical genetics, such as clinical psychology and bioethics, thus allowing an integration between the aspects of genetic diseases related to communication and bioethics and the biological and clinical ones, also considering the new techniques of genomic diagnosis [5, 12–14]. After a search in the literature databases by using the key words above mentioned, we found a few dozen articles related to general aspects of the communication skills of healthcare professionals and to genetic counselling, and about ten among them were of the last decade [12–20]. Even fewer were the studies found on prenatal counselling, as well as on communication of neonatal genetic disorders and/or single diseases, such as Down syndrome, cystic fibrosis, sickle cell anemia, thalassaemia, deafness [21–25]. A small nucleus of experts redacted the first report, summarizing all available scientific information. It was then submitted to the attention of a multidisciplinary jury, composed by representatives of the Scientific Societies and by the Presidents/Coordinators of the Parents’ Associations involved. The document thus produced was subsequently revised by an extended team of experts, made also by the Presidents and Study Groups Coordinators of the participating Scientific Societies.

## Results

The consensus of experts drafted a final document defining the recommendations, for the neonatologist or the pediatrician working in a fist level birthing center, relating to the first communication of a genetic disease or malformation syndrome diagnosis. Although there is no universal communication technique to make the informative process effective, we tried to identify a few relevant strategic principles which the neonatologist/pediatrician may use in the relationship with the family. We also summarized basic principles and the most relevant aspects on the modalities of interaction with families in a table, to create an easy tool for health care professionals to be applied in the daily care practice. We finally obtained an intersociety document, now published on the websites of the Scientific Societies involved.

## Discussion

The communication of a diagnosis of a disease with poor or disabling outcome, in which an effective therapeutic and support plan may not be proposed, is a negative event from an emotional and relational point of view. Good communication is recognized as a pivotal feature for healthcare professionals, and needed for the quality of care they provide [26]. This ability is continuously subjected to evaluation by patients, who always consider it a priority among doctor’s skills [27]. Moreover, it is documented that physicians perceive themselves more effective in communicating with patients when their clinical suspicion is confirmed by genetic (cytogenetic and/or molecular) investigations. By converse, families report that communicative effectiveness does not depend on the diagnostic definition. The importance attributed by doctors to diagnosis may reflect their well-known tendency to give value to clinical tests and to diagnostic or therapeutic procedures, while many patients give more weight to the intrinsic importance of the communication process (defined as personal utility) [27]. However, in Italy there is to date no specific training course for the medical profession, during the degree course, aimed at acquiring communication skills, nor subsequently for the neonatologist (such cultural gap, however, does not arise for medical genetics specialty, where counseling is one of the fundamental topics of training), during the Specialization School in Pediatrics. Performing simulations of communication scenarios, within the specific clinical activities of each care setting, may be useful for improving staff skills [28].

Improving the doctor-patient/family relationship may have a favorable effect on health outcomes of children and their parents, as well as reduce the phenomenon of defensive medicine and the medico-legal disputes. To implement optimal doctor-patient relationships, the principles of family-centered-care (such as, for example, collaborating with families and respecting their diversities, sharing information and providing individualized care) must be part of post-graduate training courses, policies and operational protocols of hospitals, and behaviors of neonatology and pediatric departments staff [29].

Relationships between team members and parental couple have a major impact in supporting parents’ role, especially if continuity and uniformity of medical and nursing care is guaranteed. Many behaviors of health professionals negatively considered by parents widely reflect difficulties in communication or interpersonal skills, such as a reduced amount of time dedicated to information, or a modality without sensitivity, or also an attitude excessively oriented to clinical aspects. Simple actions, such as increasing attention to the time intended for news transmission, simplifying and adapting language to the interlocutor’s profile, recognizing the positive aspects of the child, and especially the attitudes which offer support (practical and emotional) to parents’ role, may be decisive. It is essential to understand families’ point of view, because it allows to interpret their needs [30], and therefore to identify possible solutions to the most pressing problems, sometimes apparently far from doctors’ competences.

In the case of the first communication of a genetic disease diagnosis, the neonatologist/pediatrician has the aim of creating the relationship between health professionals and family. Thus, he lays the foundations to guide parents towards the clinical goal and, through a circular path, he supports and increases trust in their child, giving further strength to the care process. At the same time, he provides all the technical information on the updated clinical and scientific data of the disease which is object of the communication. The ability to fulfill these aims is partly related to the variable expertise of neonatologists/paediatricians in approaching malformation syndromes and genetic disorders, with differences depending on whether the baby is born in a first level birthing center or in a referral neonatal unit, even more if it is located in a big and/or University Hospital. However today, regardless of the clinical setting, neonatologists and pediatricians must know next generation sequencing (NGS) techniques (which simultaneously analyze millions of small DNA fragments), as they are an effective diagnostic tool increasingly used within the daily clinical activity. The current recommendation is to perform these tests, as first, in presence of neurodevelopmental disorders/intellectual disability, conditions with genetic heterogeneity (e.g. ataxia) or non-specific or unexplained clinical manifestations, also in light of their greater diagnostic efficacy compared to molecular cytogenetic analyses. NGS (which include panel sequencing and whole exome/genome sequencing, WES/WGS) changed the diagnostic process in the suspicion of rare disease, since diagnosis, unlike the traditional approach, may be reached by a single examination. However, some critical issues related to timing and indications, interpretation, ethical aspects and accessibility, as well as analytical limits need still to be resolved [31]. Furthermore, in cases with severe neonatal clinical onset, the timely achievement of a genomic diagnosis, during the period of hospitalization in the NICU, may have relevant effects on clinical management. In addition to the potential ability to modify treatments for the cases in which a cure is possible, genomic diagnosis allows a reasoned refocusing of the cure pathway, offers the possibility to reduce the suffering of newborns and to provide support to families, avoiding clinical obstinacy, disproportionate care or futile treatments. Therefore, genomic information may shift attention, in critical situations, from invasive management to a different goal, which is the relief of suffering [32]. This will be useful also for better communication and relationship with families.

In the case of a malformative picture which is evident at birth, communication is particularly challenging, especially if prenatal diagnosis is lacking. In these circumstances, the neonatologist must simultaneously fulfill, in an urgent situation, therapeutic management (stabilizing the adaptation to extrauterine life and preventing any complications), diagnostic procedures (aimed at identifying the cause of the clinical picture), and communication with the parental couple. Parents, indeed, in a context of extreme intensity and vulnerability, must be adequately and timely informed, and involved in the care process. The neonatologist is faced with pressing questions, such as: “what and why did it happen? who is responsible for it? may other problems arise? may they be cured? how will he/she be when he/she grows up? ...” , and many others will emerge in a short time. Communication with parents in the first hours after birth is then difficult and often decisive, and requires immediacy, sensitivity, experience and adequate skills [33]. The information transmitted, the verbal and non-verbal language used, the place and context in which this occurs, will have a profound impact on the family for many years [34]. Poor communication has, in fact, a long-term negative effect both on the ability to accept diagnosis and to adapt to the new situation, and on the developing relationships between parents and children. Therefore, health and psychological development of the child may be adversely affected for a long time.

There is no communication technique which may be considered effective in all circumstances. However, we may indicate 5 strategic principles:

1. Usage/empathy. It consists of the use of every element that comes from parents (language, attitude, arguments, etc. ...) for the primary aim of communication, that is the creation of the relationship between health care professionals and family. We must avoid to openly contest the parental couple: every “clash” may be a danger for the relationship, and favor mechanisms of closure and distrust.
2. Attitude of listening towards family.
3. Flexibility. The operator must adapt himself to the interlocutor’s cultural and social level, removing commonplaces and prejudices. The higher the level of preconception is, the greater the degree of stereotypy of the communicative moves increases. This is followed by lowering of the therapeutic efficacy, and removal of parents.
4. Parsimony. Clear messages, formulated with suggestive style and sparingly delivered, are easy to understand, generally effective, and get the most for the least. They must not be overly specific, full of technicalities and scientific references. Rather, it can be useful to use anecdotes, metaphors or examples, which lead to greater involvement of parents allowing them to follow suggestions and prescriptions with commitment and trust.
5. Restructuring. It means to insert the definition of a problem within other systems of meaning. It implies helping family to identify different points of view on aspects of their life experienced, until that moment, as particularly critical and painful. This is useful and functional, also in order to guide parents towards what “they can do” within the diagnostic-therapeutic and/or support plan proposed by the health care professional.

Communication is strategic if it respects these 5 principles. To formulate specific interventions it is essential to know the interlocutor, and to be able to recognize his multiple signals. To grasp and understand all the possible traces that the family may provide in the relationship, it is necessary to give all the time needed to communication. The “psychic time” of parents, which is requested for accepting a child unlike from what they imagined until that moment, is often different than that of the medical intervention. Recognizing this time and being generous with one’s own have, then, a therapeutic as well as an ethical value.

It may be useful to resort to some gimmicks to improve communication effectiveness. It may be advantageous, for example, while still maintaining objectivity, to provide first the positive aspects of a news, and then introduce the negative ones. Reversing this order decreases the positive part of the information, because the negative news, which is perceived as the most relevant, if presented first inhibits the perception of the positive one. To increase communication effectiveness, speeches must be directly reported to the interlocutor, avoiding impersonal expressions. These phrases do not recognize to families the specificity of their experience and the particularity of the interaction in which they are involved. A personalized communication takes into account the subject, and is full of references to his experience. It is preferable to use the first plural person (we), instead of the first and second singular one. In this way, it is transmitted the message of being involved in the relationship, and a greater willingness to perform specific behaviors may be obtained. A continuous and direct eye contact reflects true interest, and it guarantees active listening [35]. In Table 1 are summarized the indications for the neonatologists/pediatricians on the first communication of genetic disease/malformation syndrome diagnosis. It may be a useful and practical tool to make easier the informative process with the parents of these patients, as well as to implement the best practices in the relationship with families. Among the suggestions provided, it is particularly remarkable the relevance of the multi-specialty/multi-professional approach to these patients, even more during the difficult time of the first communication of diagnosis. The clinical geneticist and/or neonatologist or other specialist who has direct knowledge of the newborn, with competence on the disease object of the diagnosis and, if possible, the responsibility of care, may have a key role in the interaction with parents. However, since patients with genetic diseases are frequently burdened by the occurrence of malformations affecting multiple organs and systems, other specialists (e.g. pediatric surgeon) and health professionals (e.g. physiotherapist) should be involved or available. The presence of the cultural mediator is useful for those born to foreign parents. Likewise, trusted persons and representative of the reference Association (if available) must be taken into consideration within the informative process, since its initial steps. The synergy of different supporting figures with specific skills and competences, as well as a multidisciplinary management (including gynecologist, obstetrician, neonatologist, surgeon, psychologist ...), may guarantee to families a global, integrated and continuous care, and reduce the risk of disorientation or sense of abandonment. In the meantime, such approach may avoid the risks associated with the autonomous search for medical information by families. In regard to this, a guided web navigation may be crucial, since misinformation from uncertified internet can jeopardize the efforts of a correct communication.

**Table 1:** Indications for the neonatologist and the pediatrician to make easier the communication of a genetic disease and/or malformation syndrome diagnosis, and to implement the best practices in the relationship with families [36, 37]

When	• The first interview with the parents/family members should be carried out at the time of the clinical diagnosis (postponing the communication until the karyotype is available, for example in the case of Down syndrome, is considered unprofessional and intolerable by the family). • In cases of long-lasting diagnostic pathways (for example diagnosis of genetic and/or genomic diseases made by array-CGH/NGS techniques), it is necessary to share with parents the different moments and results obtained during the diagnostic process • In cases where the diagnosis is not defined, it is however possible to make a functional diagnosis, to which refer to for care program and follow-up planning.
Who	• Clinical geneticist and/or neonatologist or other specialist who has direct knowledge of the newborn, with competence on the disease object of the diagnosis and, if possible, the responsibility of care, owing to their expertise regarding the syndrome as well as in explaining methods and timing of diagnostic tests. • Genetic diseases are frequently burdened by the presence of malformations affecting multiple organs and systems and require multi-specialty/multi-professional management. Therefore, other specialists (e.g. pediatric surgeon) and health professionals (e.g. physiotherapist) should be present or available. • For those born to foreign parents the presence of the cultural mediator is useful.
With whom	• The first meeting to communicate the diagnosis should be carried out with both parents (avoiding the presence of other figures not directly involved in care). • If the mother is not in good clinical conditions, the first communication may be made with the father (with whom times and methods to inform the mother should be agreed). • The interview must be carried out in front of the newborn. His presence allows parents to be guided towards his physiological characteristics, skills, and potential. • In subsequent meetings, the involvement of family pediatrician, other specialists/health professionals, other family members/trusted persons, representative of the reference Association (if available) may be agreed. A multidisciplinary management (gynecologist, obstetrician, neonatologist, surgeon, psychologist ...), may guarantee to families a global, integrated and continuous care, and reduce the risk of disorientation or sense of abandonment.
Where	A private environment must be identified: • free from possible interruptions (by telephone, other colleagues, etc. ...); • available exclusively to parents at the end of the interview.
How	• In a proactive way, with empathy and respect. • Balance honesty and frankness (it is better a sincere “I don’t know” than incorrect or inaccurate indications, with negative repercussions on the whole life of the child and the family) with the need of parents to maintain hope for the survival of their baby. • Speak simply and clearly, using common language and minimizing jargon. • Avoid the attitude of over-describing the clinical aspects. • Refer to the child by name. • Ensure continuous and direct eye contact. • Arrange distances in the range of 50–100 cm, i.e. between “personal” and “social”, placing the chairs side by side and not in front (the interposition of the desk may be perceived as an obstacle to communication). • Accept the experience of family members and their vision of things, without directly contesting them. • Provide practical and emotional support to the process of remodeling parents’ role. • Suspend critical judgment, interpretations and “thought readings” during the interview. • Provide first the positive aspects of a news, and then introduce the negative ones. • Use the first plural person (we) instead of the first or second singular one. • Avoid impersonal expressions, and directly refer to the experience of parents. • Assess parents’ knowledge, their cultural background, and ethical visions, as well as the ability to understand what is explained to them. • Facilitate questions and requests from parents with opening speeches.
How long	• First meeting without time limits, however avoiding overflowing interviews that lose effectiveness and increase the risk of misunderstandings. • Provide more meetings, as needed, with different methods and professionals. • In any case, dedicate all the time you need to communication. The “psychic time” of parents, necessary for accepting a child unlike from what they imagined until that moment, is often different than that of the medical intervention. Recognizing this time and being generous with one’s own have, then, a therapeutic as well as an ethical value.
What	• Provide updated information on the main characteristics of the newborn’s disease, reporting only the most frequent complications, without listing the rarest or most unlikely ones (which indicate theoretical knowledge, but which do not give any benefit to the family). • Explain genetic and/or genomic diagnostic procedures, documenting the clinical indications. • Perform a physical examination of the newborn together with the parents, underlining his physiological features. • Formulate an individualized and realistic prognosis (not theoretical and applicable in a generic way to other patients with the same disease), and perform a reproductive counseling. • Describe the care program and provide information on follow-up, reference center and local services.
With which supports/helps	• Informatic and/or written material (brochures, information booklets also relating to the presentation of the reference Association), guided web navigation in order to avoid that misleading information can disrupt the correct care or adaptation process. • Parents/families’ associations, institutions.

The pediatrician-parents relationship is a communicative situation in which the goal is common and shared. The strategic mandate of the pediatrician consists of 1) leading the relationship; 2) accompanying parents towards the clinical goal; 3) keeping always high motivation and trust; 4) ensuring good levels of compliance and managing any unexpected events.

Sometimes fulfilling this task is for the pediatrician a simple, spontaneous and immediate activity. In fact, with many parents it is not necessary to consciously assume a strategic attitude. These are situations in which communication simply flows and generates balances functional to the treatment, which facilitate doctor’s work. Other times, however, the relationship with family members is critical, and care may be affected. In these cases, both patient’s well-being and that of the healthcare worker are at risk. This happens, for example, with the parents of children with genetic disease, with whom communication is more difficult. Indeed, they are discouraged and question the role and the therapeutic “power” of those who cover it. The pediatrician, in these cases, will be strategic if he is able to behave not only as an expert clinician, but also as a process expert. The latter knows how to pick up signals, even the weak ones, and uses them to promote the therapeutic relationship, to generate behaviors in families that are tuned to the achievement of the clinical goal.

## Conclusions

The neonatologist/pediatrician is often the first to observe complex syndromic pictures, not always identified in the prenatal period. He must be aware of all aspects around the genetic diseases, including communication, bioethics, and law [38–44] in view of a multidisciplinary care of the affected newborns and children.

A genetic diagnosis allows the families to feel less fragile, regardless of the benefit that it may have on the clinical management. NGS techniques allowed to provide early genetic counseling [45–48]. When promptly obtained, they may support and guide clinicians to the most appropriate clinical management and communicative approach, avoiding futile and/or disproportionate treatments [32], as well as further unnecessary separations between children and their parents [49].

This consensus provides neonatologists and pediatricians with practical recommendations around the first communication of a genetic disease/malformation syndrome diagnosis. The indications reported may be a useful tool to make easier the informative process, and to implement the best practices in the relationship with families. Communication must not be a marginal or secondary clinical activity. By converse, it requires a careful attention of operators, also considering its clinical and socioeconomic implications on the short and long term. Indeed, a better doctor-patient/family interaction may improve several health outcomes of the child and his family. In addition, it may also reduce the legal disputes with parents and the phenomenon of defensive medicine.

## Data Availability

The datasets used and analyzed during the current study are available from the corresponding author on reasonable request.

## Abbreviations

CGH: Comparative genomic hybridization
NGS: Next generation sequencing
NICU: Neonatal intensive care unit
USA: United States of America
WES: Whole exome sequencing
WGS: Whole genome sequencing
